# Spicy food consumption is associated with abdominal obesity among Chinese Han population aged 30–79 years in the Sichuan Basin: a population-based cross-sectional study

**DOI:** 10.1186/s12889-022-14293-4

**Published:** 2022-10-10

**Authors:** Xianxian Yang, Wenge Tang, Deqiang Mao, Xiang Liu, Wen Qian, Yingxue Dai, Liling Chen, Xianbin Ding

**Affiliations:** 1 Department of Non-Communicable Disease Control and Prevention, Chongqing Center for Disease Control and Prevention, Chongqing, 400042 China; 2 West China School of Public Health and West China Fourth Hospital, Sichuan University, 610041 Sichuan, China; 3 Chengdu Center for Disease Control and Prevention, Chengdu, 610047 China

**Keywords:** Spicy food consumption, Abdominal obesity, Waist circumference, Han population

## Abstract

**Background:**

Few animal experiments and volunteer-based intervention studies have showed a controversial effect of spicy foods on abdominal obesity. We aimed to examine the association between spicy food frequency, spicy flavor, and abdominal obesity among Chinese Han population in the Sichuan Basin which area eating spicy foods relatively often.

**Methods:**

A cross-sectional analysis was conducted using the Sichuan Basin baseline data from the China Multi-Ethnic Cohort (CMEC) study, including data from electronic questionnaires, anthropometric measurements and blood sample collection. A total of 40,877 adults (22,503 females) aged 30–79 years were included in the final analysis. Multivariable logistic regression yielded adjusted odds ratios (ORs) and 95% confidence intervals (CIs) for abdominal obesity associated with the strength of spicy flavor and frequency of spicy food intake.

**Results:**

The prevalence of daily spicy food eating was 47.3% in males and 52.7% in females, the percentages of abdominal obesity were 52.3%, 48.8%, 51.6% and 55.5% in the spicy food intake subgroups of never, 1–2 days/week, 3–5 days/week and 6–7 days/week, respectively. Compared with males who never consumed spicy food, the adjusted ORs (95% CIs) in the 1–2 days/week, 3–5 days/week and 6–7 days/week subgroups were 1.21 (1.09, 1.34), 1.35 (1.21, 1.51), and 1.35 (1.25, 1.47), respectively (*P*_*trend*_ < 0.001). The corresponding odds ratios for females were 0.95 (0.87, 1.05), 1.14 (1.03, 1.26), and 1.25 (1.16, 1.35), respectively (*P*_*trend*_ < 0.001). Similarly, compared with no spicy flavor, the adjusted ORs (95% CIs) of mild, middle, and strong spicy strength for abdominal obesity in males were 1.27 (1.17, 1.38), 1.51 (1.37, 1.67), and 1.36 (1.11, 1.67) respectively (*P*_*trend*_ < 0.001). The corresponding odds ratios for females were 1.14 (1.06, 1.23), 1.27 (1.15, 1.40), and 1.32 (1.06, 1.65), respectively (*P*_*trend*_ < 0.001).

**Conclusions:**

The data indicated that spicy food consumption was a risk factor for abdominal obesity among Chinese adult population in the Sichuan Basin. The results need to be approved by large cohort studies.

## Background

Obesity is associated with the common chronic diseases, including hypertension, type 2 diabetes, coronary heart disease, and certain types of cancer, and is considered as the fifth leading risk factors for mortality globally [1–7]. Obesity is classified as general obesity and abdominal obesity. Compared with Western societies, Asian population have a relatively lower body mass index (BMI) but are predisposed to central or abdominal obesity [8–11]. In particular, abdominal obesity has a close relationship with central fat localization and cardiovascular disease, independently of general obesity [12, 13]. Studies have shown that abdominal adipose tissue is more metabolically active and is the key determinant of metabolic abnormalities that contribute to obesity-related disease risk [12, 14, 15]. In recent years, the prevalence of abdominal obesity is increasing dramatically in China [16]. According to the data obtained in the China Health and Nutrition Survey (CHNS) in 2011, the age-adjusted prevalence of abdominal obesity in China was 35.3% in men and 51.7% in women, and from 1993 to 2011, mean waist circumference (WC) significantly increased among men and women [17].

Spices are recognized as an essential part of culinary cultures, with a long history of using for flavoring, coloring and preserving food, as well as for medicinal purposes in the world [18, 19]. Spicy intake is substantial higher in ancient Asian than European countries, especially in India and China [20, 21]. In certain regions of China such as Hunan and Sichuan, almost a third of adults consume spicy food daily, including chili [22]. In the past decades, although several previous studies have indicated that the consumption of spicy food affects obesity-related human health outcomes, such as hypertension [23], irritable bowel syndrome [24], lipid disorders [25], cancers [26] and even mortality [27]. As the most practical and simplest index, WC can provide information on the distribution of body fat and is strongly correlated with central fat localization [28, 29]. The association between spicy food consumption and obesity has received considerable attention in recent years [30–32]. Sun and colleagues [30] conducted in five urban and five rural areas of China provided evidence that the frequency of spicy food consumption was positively associated with WC. However, half a million participants come from different regions in the study, and with the different education level, economic development, behavior factors, food choices and dietary patterns.

Therefore, it has more practical guiding significance to explore the associations of spicy food and risk of obesity in different regions and people with different dietary habits. Sichuan Basin is located in the southwest of China, due to the humid climate, residents have formed a spicy food habit for a long time. Hence the aim of this study was to examine the relationship between spicy food consumption and abdominal obesity in the Sichuan Basin using baseline data from the CMEC study in a large-scale Han Chinese population in the southwest China.

## Materials and methods

### Study design and participants

This cross-sectional study based on the CMEC study. Detailed information about the CMEC study design, survey methods and population have been described elsewhere [33, 34]. The data utilized in the current study were obtained from Sichuan Basin region, two (Chongqing and Sichuan provinces) of the 5 regions included in the CMEC study. In brief, 44,900 Chinese Han participants aged 30–79 years were recruited and participated in the baseline survey during June 2018 to February 2019. This population-based survey was carried out in 18 districts/counties by multistage, stratified cluster sampling from Chongqing (the district and county are of the same administrative level in Chongqing), and Sichuan provinces, including Yuzhong District, Jiulongpo District, Nanan District, Banan District, Changshou District, Jiangjin District, Hechuan District, Qijiang District, Dazu District, Tongnan District, Rongchang District, Wulong District, Fengdu County, Chenghua Distric, Qingbaijiang Distric, Wuhou District, Pidu District and Jianyang city.

**Fig. 1:**
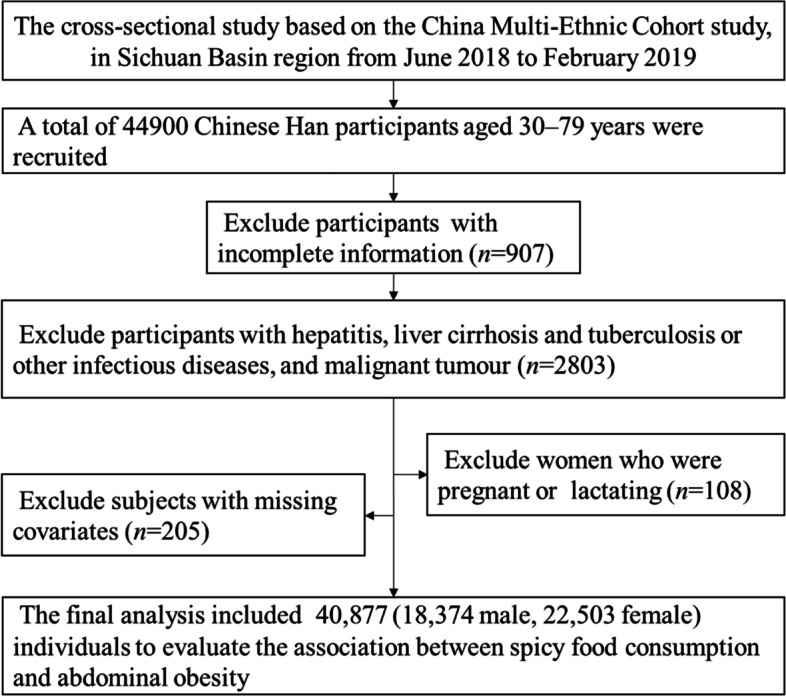
Data cleaning flow chart

A total of 43,993 people aged between 30 and 79 years have completed the questionnaire and anthropometric measurements and with complete information for spicy food intake and WC measurements were recruited into the current study. To better evaluate the relationship between spicy food consumption and abdominal obesity, 2803 participants with hepatitis, liver cirrhosis and tuberculosis or other infectious diseases, and malignant tumour, and 108 women who were pregnant or lactating, and 205 subjects missing covariates were excluded. After these exclusions, a total of 40,877 (18,374 male, 22,503 female) participants were included in the current analysis (Fig. 1). All participants provided written informed consent prior to the survey. The study was approved by the Sichuan University Medical Ethical Review Board (K2016038) and the Research Ethics Committee of Chongqing Center for Disease Control and Prevention (2017(001)).

### Assessment of spicy food consumption

Frequency of spicy food consumption was assessed through the question “During the past month, how often did you have spicy food in one week?” Answer options included: “Never or almost never”, “only occasionally” (i.e., less than once weekly), “1–2 days per week”, “3–5 days per week”, and “daily or almost every day”. In analyses, those who chose “Never or almost never” or “only occasionally” were combined into one group (i.e., Never and only occasionally). Among those who ate spicy food more than once a week, participants were asked at what age they started to eat spicy food, and the strength of spicy food eating they usually preferred. Participants were also asked “How much do you like the spicy flavor in your food?” Answer options included: “Never”, “Mild”, “Moderate”, and “Strong”. So the strength of spicy food eating was categorized as never, mild, moderate and strong. Further, years of eating spicy food-to-current age ratio is used as a measure of a life-long burden of spicy food consumption.

### Assessment of covariates

We used a tablet computer with a self-developed application (CMEC App) to collect the questionnaire information. The information was collected by a face-to-face interview implemented by well-trained interviewers who were typically local college students with medical backgrounds. The electronic questionnaire included detailed questions on socio-demographic status (age, sex, study area, education level, marital status, and annual household income), lifestyle factors (tobacco use, alcohol use, physical activity, the total daily energy intake), medical history and health status, and, among women, menopause status.

For the baseline survey, dietary information was assessed using a quantitative food frequency questionnaire (FFQ) based on the Chinese Dietary Guidelines and the eating habits of Southwestern Chinese, covering 13 major food groups: rice, wheat products, coarse grain, tubers, red and processed meats, poultry and processed, fish/sea food, eggs, dairy products, fresh vegetables, fresh fruits, and soybean products. For each food group, participants were required to report the quantity (average grams per meal according to standard serving size mould) and frequency (four frequency categories ranged from how many times per day to year) that they consumed during the past 12 months. We also asked information about alcohol, tea, Sugar Sweetened Beverages (SSBs), cooking oil and salt in separate sections. In particular, daily alcohol consumption was calculated as grams of pure alcohol according to alcohol type, amount drunk and frequency [35]. From the above FFQ, we estimated the total daily energy intake according to the China food exchange lists [36] and the 2018 China food composition tables (standard edition) [37] for each individual. In addition, we conducted the repeated FFQ and 24-h dietary recall (24HDRs) to assess the reproducibility and validity of the baseline FFQ. Regarding reproducibility, intraclass correlation coefficients (ICC) for food groups ranged from 0.15 for fresh vegetables to 0.67 for alcohol. Regarding validity, de-attenuated Spearman rank correlation coefficients for food groups ranged from 0.10 for soybean products to 0.66 for rice. More details are provided in our previous study [34].

For assessment of tobacco consumption, participants were asked how often they had smoked tobacco during the previous 12 months at the time of the survey, and those who had not smoked weekly were asked if there was a period of at least a year prior to that when they had smoked some tobacco on most days or daily. Participants were classified into three main smoking categories: 1) nonsmokers; 2) ex-smokers; 3) current smokers. Total physical activity was converted into metabolic equivalent of task hours per day (MET-hours/day) based on transportation, occupation, housework, and leisure time activities as described in previous studies [34, 38].

Body weight with light clothing was measured to the nearest 0.1 kg using a weight measurement device. Height was measured to the nearest 0.1 cm without shoes using a standard right-angle device and a fixed measurement tape. The body mass index (BMI) was calculated as weight divided by height squared (kg/m^2^). Participants were classified according to BMI following the recommendation of the WHO.

### Assessment of outcomes

WC was measured to the nearest 0.1 cm at a point midway between the lowest rib and the iliac crest in a horizontal plane using a non-elastic tape. All measurements were made by trained staff using a standard protocol and instruments. Abdominal obesity was defined as a WC ≥ 85.0 cm for men and ≥ 80.0 cm for women according to the guideline for prevention and control of overweight and obesity in Chinese adults [39].

### Statistical analyses

Statistical analyses were performed using the Statistical Program for Social Sciences (SPSS) version 25.0 (SPSS Inc., Chicago, IL, USA). All analyses were performed separately by gender groups. According to the frequency of spicy food intake, descriptive statistics were presented as number (percentage) or median (Q1, Q3 percentiles) for categorical or continuous variables, respectively. Chi square (χ^2^) test and Kruskal–Wallis H test were performed to compare the differences in covariates among various intake frequency groups. Logistic regression analysis was used to estimate the association between the frequency and strength of spicy food intake and risk of abdominal obesity after adjusting for potential confounding variables based on the odds ratios (OR) and 95% confidence interval (CI). Participants who never consumed spicy food were considered as the reference group. Potential confounding factors, including sociodemographic status and lifestyle factors were adjusted for in different models. A set of models were used: model 1 adjusted for age, gender. Model 2: further adjusted for marriage status, education, household income, study area. Model 3: further adjusted for tobacco use, alcohol use, total energy intake and physical activity. Model 4: adjusted as for model 1 minus gender. Model 5: adjusted as for model 2 minus gender. Model 6: adjusted as for model 3 minus gender. Model 7: adjusted as for model 2 minus gender, plus menopause status. Model 8: adjusted as for model 3 minus gender, plus menopause status. Stratified subgroup analyses were conducted according to the demographic characteristics to detect the associations of spicy food consumption on the risk of abdominal obesity. All statistical tests were two sided and a *P* value < 0.05 was considered statistical significant.

## Results

### General characteristics

Table 1 summarizes the general characteristics of the participants according to the spicy food frequency and strength. The median age of 40,877 subjects (18,374 males, 22,503 females) was 49.7 (42.8, 61.0) years old, and the median value of waist circumference was 86.0 (80.0, 92.0) cm for males and 80.0 (73.0, 86.0) cm for females. The daily spicy food consumers accounted for 47.3% in males and 52.7% in females, and most of them were from urban areas for 58.8%. The prevalence of abdominal obesity among participants who consumed spicy food never, 1–2 days/week, 3–5 days/week, and 6–7 days/week, were 52.3%, 48.8%, 51.6% and 55.5%, respectively. There were significant differences in the characteristics of age, gender, marital status, education level, annual household income, study area, tobacco and pure alcohol use, red and processed meats, poultry and processed, fresh vegetables, fresh fruits, total energy intake, physical activity, BMI, WC, abdominal obesity and age started to eat spicy food between the spicy food intake frequency subgroups, strength of spicy food intake subgroups respectively (*p* < 0.05). Moreover, the higher the frequency and strength of spicy food eating, the higher the physical activity level, as well as total energy intake and the prevalence of current smokers. Among those who ate spicy food regularly (participants who ate spicy food more than 1–2 days/week), the more frequently who ate spicy food, and the stronger the intensity of spicy flavor who preferred, and the longer the duration of eating spicy food.

**Table 1:** Characteristics of the participants according to the spicy flavor and spicy food intake frequency

**Characteristics**	**Total (*n* = 40877)**	**Spicy food intake frequency, n(%)**									***P***
		**Never (*n* = 8372)**			**1–2 days/week (*n* = 6538)**		**3–5 days/week (*n* = 4924)**		**6–7 days/week (*n* = 21043)**		
**Ages (years)**	49.7 (42.8, 61.0)	54.4 (45.9, 65.5)			47.7 (40.1, 55.8)		46.6 (38.8, 54.3)		49.2 (42.3, 58.1)		< 0.001
**Gender**											< 0.001
** Male**	18374 (44.9)	3481 (41.6)			2738 (41.9)		2203 (44.7)		9952 (47.3)		
** Female**	22503 (55.1)	4891 (58.4)			3800 (58.1)		2721 (55.3)		11091 (52.7)		
**Marriage status**											< 0.001
** Married/cohabitation**	36365 (89.0)	7223 (86.3)			5798 (88.7)		4371 (88.8)		18973 (90.2)		
** Unmarried/separated/divorced/widowed**	4512 (11.0)	1149 (13.7)			740 (11.3)		553 (11.2)		2070 (9.8)		
**Education level**											< 0.001
** Primary school and below**	13110 (32.1)	3738 (44.7)			1675 (25.6)		1060 (21.5)		6637 (31.5)		
** Middle school**	13133 (32.1)	2482 (29.6)			2097 (32.1)		1637 (33.2)		6917 (32.9)		
** High school**	7402 (18.1)	1144 (13.7)			1316 (20.1)		1052 (21.4)		3890 (18.5)		
** College or university and above**	7232 (17.7)	1008 (12.0)			1450 (22.2)		1175 (23.9)		3599 (17.1)		
**Annual household income (CNY)**											< 0.001
** < 12000**	4118 (17.1)	1355 (16.2)			591 (9.0)		356 (7.2)		1816 (8.6)		
** 12000–19999**	4944 (12.1)	1315 (15.7)			765 (11.7)		503 (10.2)		2361 (11.2)		
** 20000–59999**	14610 (35.7)	3033 (36.2)			2287 (35.0)		1720 (34.9)		7570 (36.0)		
** 60000–99999**	8686 (21.3)	1503 (18.0)			1460 (22.3)		1162 (23.7)		4561 (21.7)		
** ≥ 100000**	8519 (20.8)	1166 (13.9)			1435 (22.0)		1183 (24.0)		4735 (22.5)		
**Study area**											< 0.001
** Rural**	16356 (40.0)	3740 (44.7)			2265 (34.6)		1685 (34.2)		8666 (41.2)		
** Urban**	24521 (60.0)	4632 (55.3)			4273 (65.4)		3239 (65.8)		12377 (58.8)		
**Tobacco use**											< 0.001
** No smokers**	29032 (71.0)	6651 (79.5)			4941 (75.6)		3487 (70.8)		13953 (66.3)		
** Ex-smokers**	2621 (6.4)	506 (6.0)			393 (6.0)		302 (6.1)		1420 (6.8)		
** Current smokers**	9224 (22.6)	1215 (14.5)			1204 (18.4)		1135 (23.1)		5670 (26.9)		
**Alcohol use (grams per week)**	5 (0, 5)	0 (0, 0)			0 (0, 5)		0 (0, 5)		0 (0, 5)		< 0.001
**Red and processed meats (grams per week)**	500 (300, 700)	350 (200, 700)			350 (210, 700)		450 (300, 700)		700 (350, 750)		< 0.001
**Poultry and processed (grams per week)**	70 (23.3, 150)	46.7 (11.7,105)			60 (23.3, 140)		70 (31.2, 150)		70 (24, 150)		< 0.001
**Fresh vegetables (grams per week)**	2100 (1400, 3500)	2100 (1400, 3500)			2100 (1400, 3500)		2100 (1400, 3500)		2100 (1400, 3500)		< 0.001
**Fresh fruits (grams per week)**	700 (300, 1400)	600 (150, 1400)			700 (350, 1400)		700 (350, 1400)		700 (300, 1400)		< 0.001
**Total energy intake (kcal/day)**	1644.8 (1318.1, 2054.4)	1442.5 (1152.2, 1809.9)			1481.8 (1205.9, 1820.6)		1505.8 (1238.8, 1860.4)		1554.5 (1272.4, 1901.7)		< 0.001
**Physical activity (METs-h/day)**	28.1 (17.7, 38.6)	23.6 (14.7, 36.0)			27.9 (17.6, 37.0)		28.0 (18.7, 37.3)		27.6 (17.6, 37.9)		< 0.001
**Menopause **^**a**^											< 0.001
** No**	11986 (53.3)	1881 (38.5)			2256 (59.4)		1772 (65.1)		6077 (54.8)		
** Yes**	10517 (46.7)	3010 (61.5)			1544 (40.6)		949 (34.9)		5014 (45.2)		
**BMI (kg/m**^**2**^**)**	24.3 (22.2, 26.6)	23.96 (22.0, 26.3)			23.5 (21.6, 25.6)		23.5 (21.6, 25.8)		24.07 (22.0, 26.5)		< 0.001
**WC (cm)**											
** Male**	86.0 (80.0, 92.0)	85.0 (79.0, 90.0)			86.0 (80.0, 91.0)		87.0 (80.0, 9.02)		86.5 (80.0, 92.0)		< 0.001
** Female**	80.0 (73.0, 86.0)	80.0 (74.0, 86.0)			78.0 (72.0, 84.0)		78.0 (73.0, 85.0)		80.0 (74.0, 87.0)		< 0.001
**Abdominal obesity**											< 0.001
** No**	19081 (46.7)	3989 (47.7)			3347 (51.2)		2383 (48.4)		9362 (44.5)		
** Yes**	21796 (53.3)	4383 (52.3)			3191 (48.8)		2541 (51.6)		11681 (55.5)		
**Strength of spicy food intake**											< 0.001
** Never**	8372 (20.5)	8372 (100.0)			0 (0.0)		0 (0.0)		0 (0.0)		
** Mild**	24814 (60.7)	0 (0.0)			5780 (88.4)		3943 (80.1)		15091 (71.7)		
** Moderate**	6848 (16.8)	0 (0.0)			690 (10.6)		920 (18.7)		5238 (24.9)		
** Strong**	843 (2.0)	0 (0.0)			68 (1.0)		61 (1.2)		714 (3.4)		
**Age started to eat spicy food**	5 (5, 15)	NA			10 (5, 20)		8 (5, 17)		5 (5, 1)		< 0.001
**Years of eating spicy food**	40 (30, 49)	NA			35 (26, 45)		36 (27, 44)		41 (32, 50)		< 0.001
**Characteristics**	**Total (*n* = 40877)**		**Strength of spicy food intake, n (%)**							***P***	
			**Never (*n* = 8372)**	**Weak (*n* = 24814)**		**Moderate (*n* = 6848)**		**Strong (*n* = 843)**			
**Ages (years)**	49.7 (42.8, 61.0)		54.4 (45.9, 65.5)	48.8 (41.8, 57.4)		46.9 (38.1, 54.5)		49.9 (44.4, 60.4)		< 0.001	
**Gender**										< 0.001	
** Male**	18374 (45.0)		3481 (41.6)	10633 (42.9)		3802 (55.5)		458 (54.3)			
** Female**	22503 (55.0)		4891 (58.4)	14181 (57.1)		3046 (44.5)		385 (45.7)			
**Marriage status**										< 0.001	
** Married/cohabitation**	36365 (89.0)		7223 (86.3)	22267 (89.7)		6151 (89.8)		724 (85.9)			
** Unmarried/Separated/divorced/widowed**	4512 (11.0)		1149 (13.7)	2547 (10.3)		697 (10.2)		119 (14.1)			
**Education level**										< 0.001	
** Primary school and below**	13110 (32.1)		3738 (44.6)	7424 (29.9)		1602 (23.4)		346 (41.0)			
** Middle school**	13133 (32.1)		2482 (29.7)	8240 (33.2)		2154 (31.4)		257 (30.5)			
** High school**	7402 (18.1)		1144 (13.7)	4605 (18.6)		1518 (22.2)		135 (16.0)			
** College or university and above**	7232 (17.7)		1008 (12.0)	4545 (18.3)		1574 (23.0)		105 (12.5)			
**Annual household income (CNY)**										< 0.001	
** < 12000**	4118 (10.1)		1355 (16.2)	2102 (8.5)		546 (8.0)		115 (13.6)			
** 12000–19999**	4944 (12.1)		1315 (15.7)	2855 (11.5)		663 (9.6)		111 (13.2)			
** 20000–59999**	14610 (35.7)		3033 (36.2)	9032 (36.4)		2260 (33.0)		285 (33.8)			
** 60000–99999**	8686 (21.3)		1503 (18.0)	5468 (22.0)		1546 (22.6)		169 (20.1)			
** ≥ 100,000**	8519 (20.8)		1166 (13.9)	5357 (21.6)		1833 (26.8)		163 (19.3)			
**Study area**										< 0.001	
** Rural**	16356 (40.0)		3740 (44.7)	9755 (39.3)		2500 (36.5)		361 (42.8)			
** Urban**	24521 (60.0)		4632 (55.3)	15059 (60.7)		4348 (63.5)		482 (57.2)			
**Tobacco use**										< 0.001	
** No smokers**	29032 (71.0)		6651 (79.5)	18004 (72.5)		3940 (57.5)		437 (51.8)			
** Ex-smokers**	2621 (6.4)		506 (6.0)	1579 (6.4)		485 (7.1)		51 (6.1)			
** Current smokers**	9224 (22.6)		1215 (14.5)	5231 (21.1)		2423 (35.4)		355 (42.1)			
**Alcohol use (grams per week)**	5 (0, 5)		0 (0, 0)	0 (0, 5)		0 (0, 5)		0 (0, 5)		< 0.001	
**Red and processed meats (grams per week)**	500 (300, 700)		350 (200,700)	500 (300, 700)		700 (350, 1050)		700 (350, 1400)		< 0.001	
**Poultry and processed (grams per week)**	70 (23.3, 150)		46.7 (11.7, 105)	70 (23.3, 150)		100 (35, 160)		70 (23.3, 150)		< 0.001	
**Fresh vegetables (grams per week)**	2100 (1400, 3500)		2100 (1400, 3500)	2100 (1400, 3500)		2100 (1400, 3500)		2100 (1400, 3500)		< 0.001	
**Fresh fruits (grams per week)**	700 (300, 1400)		600 (150, 1400)	700 (350, 1400)		700 (280, 1400)		600 (116.7, 1400)		< 0.001	
**Total energy intake (kcal/day)**	1644.8 (1318.2, 2054.4)		1442.5 (1152.2, 1809.9)	1525.8 (1247.9, 1873.2)		1555.4 (1248.0, 1906.7)		1598.6 (1263.1, 1963.0)		< 0.001	
**Physical activity(METs-h/day)**	28.1 (17.7, 38.6)		23.6 (14.7, 36.0)	27.5 (17.6, 37.6)		28.6 (18.8, 37.7)		27.2 (18.7, 38.8)		< 0.001	
**Menopause **^**a**^										< 0.001	
** No**	11986 (53.3)		1881 (38.5)	7947 (56.0)		1961 (64.4)		197 (51.2)			
** Yes**	10517 (46.7)		3010 (61.5)	6234 (44.0)		1085 (35.6)		188 (48.8)			
**BMI (kg/m**^**2**^**)**	24.3 (22.2, 26.6)		24.0 (22.0, 26.3)	23.9 (21.8, 26.2)		23.8 (21.8, 26.1)		24.4 (22.4, 27.0)		< 0.001	
**WC (cm)**											
** Male**	86.0 (80.0, 92.0)		85.0 (79.0, 90.0)	86.0 (80.0, 92.0)		87.0 (81.0, 93.0)		86.2 (80.0, 93.0)		< 0.001	
** Female**	80.0 (73.0, 86.0)		80.0 (74.0, 86.0)	79.4 (73.0, 86.0)		79.0 (73.0, 85.0)		81.5 (74.0, 88.0)		< 0.001	
**Abdominal obesity**										< 0.001	
** No**	19081 (46.7)		3989 (47.7)	11689 (47.1)		3043 (44.4)		360 (42.7)			
** Yes**	21796 (53.3)		4383 (52.3)	13125 (52.9)		3805 (55.6)		483 (57.3)			
**Age started to eat spicy food**	5 (5, 15)		NA	5 (5, 15)		5 (5, 12)		5 (5, 12)		< 0.001	
**Years of eating spicy food**	40 (30, 49)		NA	39 (29, 49)		38 (29, 48)		43 (35, 51)		< 0.001	

Continuous data were described as the median (Q1, Q3), and statistical significance was assessed by the Wilcoxon rank sum test. Categorical data were summarized as percentages (%), and statistical significance was assessed by a chi-square test

^a^ Only in women

*CNY* China Yuan, *MET* metabolic equivalent of task, *BMI* body mass index, *WC* waist circumference

### Association between spicy food consumption and abdominal obesity

Table 2 shows the ORs of spicy food intake frequency for abdominal obesity in males and females. Compared with never having spicy food, the adjustment for potential confounders (age, gender, marriage status, education, household income, study area, tobacco use, alcohol use, red and processed meats, poultry and processed, fresh vegetables, fresh fruits, total energy intake and physical activity), the ORs (95% CIs) of 1–2 days/week, 3–5 days/week and 6–7 days/week subgroups were 1.04 (0.97, 1.11), 1.21 (1.13, 1.31), and 1.30 (1.24, 1.38) respectively. The adjusted OR (95% CI) was 1.10 (1.08, 1.12) (*P*_*trend*_ < 0.001) for each level increment in the frequency of spicy food intake. When the association was explored separately among males, the corresponding ORs (95% CI) were 1.21 (1.09, 1.34), 1.35 (1.21, 1.51), and 1.35 (1.25, 1.47), respectively. The adjusted OR (95% CI) was 1.10 (1.07, 1.12) (*P*_*trend*_ < 0.001) for each level increment in the frequency of spicy food intake. When the association was explored separately among females, the corresponding ORs (95% CI) were 0.95 (0.87, 1.05), 1.14 (1.03, 1.26), and 1.25 (1.16, 1.35), respectively. The adjusted OR (95% CI) was 1.10 (1.07, 1.12) (*P*_*trend*_ < 0.001) for each level increment in the frequency of spicy food intake.

**Table 2:** ORs (95% CIs) of spicy food intake frequency for abdominal obesity

**Variables**	**Frequency of spicy food intake**					
	**Never**	**1–2 days /week**	**3–5 days/week**	**6–7 days/week**	**Each level increment**	***P***_***trend***_
**Total**						
** Abdominal obesity (cases/total, n)**	4383/8372	3191/6538	2541/4924	11681/21043		
** Model 1**	1.00	1.03 (0.96, 1.10)	1.19 (1.11, 1.28)	1.31 (1.24, 1.38)	1.10 (1.08, 1.12)	< 0.001
** Model 2**	1.00	1.04 (0.98, 1.12)	1.22 (1.13, 1.31)	1.31 (1.24, 1.38)	1.10 (1.08, 1.12)	< 0.001
** Model 3**	1.00	1.04 (0.97, 1.11)	1.21 (1.13, 1.31)	1.30 (1.24, 1.38)	1.10 (1.08, 1.12)	< 0.001
**Male**						
** Abdominal obesity ( cases/total, n)**	1748/3481	1543/2738	1306/2203	5870/9952		
** Model 4**	1.00	1.29 (1.16, 1.42)	1.45 (1.30, 1.62)	1.43 (1.32, 1.55)	1.11 (1.08, 1.14)	< 0.001
** Model 5**	1.00	1.29 (1.11, 1.36)	1.38 (1.23, 1.54)	1.39 (1.28, 1.50)	1.10 (1.08, 1.13)	< 0.001
** Model 6**	1.00	1.21 (1.09, 1.34)	1.35 (1.21, 1.51)	1.35 (1.25, 1.47)	1.10 (1.07, 1.12)	< 0.001
**Female**						
** Abdominal obesity ( cases/total, n)**	2635/4891	1648/3800	1235/2721	5811/11091		
** Model 4**	1.00	0.89 (0.81, 0.97)	1.05 (0.95, 1.16)	1.23 (1.15, 1.33)	1.09 (1.07, 1.12)	< 0.001
** Model 7**	1.00	0.96 (0.88, 1.05)	1.15 (1.04, 1.27)	1.27 (1.18, 1.36)	1.10 (1.07, 1.12)	< 0.001
** Model 8**	1.00	0.95 (0.87, 1.05)	1.14 (1.03, 1.26)	1.25 (1.16, 1.35)	1.10 (1.07, 1.12)	< 0.001

Model 1: adjusted for age (continuous), gender

Model 2: age (continuous), gender, marriage status (married/cohabitation, unmarried or separated or divorced or widowed), education level(primary school and below, middle school, high school, college or university and above), annual household income (< 12,000 CNY, 12,000–19,999 CNY, 20,000–59,999 CNY, 60,000–99,999 CNY, ≥ 100,000 CNY), study area (rural, urban)

Model 3: adjusted as for model 2 plus tobacco use (no smokers, ex-smokers, current smokers), alcohol use (continuous), red and processed meats (continuous), poultry and processed (continuous), fresh vegetables (continuous), fresh fruits (continuous), total energy intake (continuous) and physical activity (continuous)

Model 4: adjusted as for model 1 minus gender

Model 5: adjusted as for model 2 minus gender

Model 6: adjusted as for model 3 minus gender

Model 7: adjusted as for model 2 minus gender, plus menopause

Model 8: adjusted as for model 3 minus gender, plus menopause

After adjusting for potential confounders in model 4, compared with never preference for spicy flavor, the adjusted ORs (95% CIs) of mild, middle, and strong for abdominal obesity were 1.19 (1.13, 1.26), 1.40 (1.31, 1.50), and 1.29 (1.11, 1.49), respectively. The adjusted OR (95% CI) was 1.16 (1.12, 1.19) (*P*_*trend*_ < 0.001) for each level increment in the strength of spicy food intake. When the association was explored separately among males, the corresponding ORs (95% CI) were 1.27 (1.18, 1.37), 1.51 (1.37, 1.67), and 1.36 (1.11, 1.67), respectively. The adjusted OR (95% CI) was 1.19 (1.14, 1.24) (*P*_*trend*_ < 0.001) for each level increment in the strength of spicy food intake. When the association was explored separately among females, the corresponding ORs (95% CI) were 1.14 (1.06, 1.23), 1.27 (1.15, 1.40), and 1.32 (1.06, 1.65), respectively. The adjusted OR (95% CI) was 1.12 (1.07, 1.17) (*P*_*trend*_ < 0.001) for each level increment in the strength of spicy food intake (Table 3).

**Table 3:** ORs (95% CIs) of spicy food intake strength for abdominal obesity

**Variables**	**Strength of spicy food intake**					
	**Never**	**Mild**	**Moderate**	**Strong**	**Each level increment**	***P***_***trend***_
**Total**						
** Abdominal obesity (cases/total, n)**	4383/8372	13125/24814	3805/6848	483/843		
** Model 1**	1.00	1.19 (1.13, 1.25)	1.37 (1.29, 1.47)	1.30 (1.12, 1.50)	1.15 (1.12, 1.18)	< 0.001
** Model 2**	1.00	1.20 (1.14, 1.26)	1.40 (1.31, 1.50)	1.28 (1.11, 1.48)	1.15 (1.12, 1.19)	< 0.001
** Model 3**	1.00	1.19 (1.13, 1.26)	1.40 (1.31, 1.50)	1.29 (1.11, 1.49)	1.16 (1.12, 1.19)	< 0.001
**Male**						
** Abdominal obesity (cases/total, n)**	1748/3481	6105/10633	2348/3802	266/458		
** Model 4**	1.00	1.34 (1.24, 1.45)	1.61 (1.47, 1.77)	1.38 (1.13, 1.68)	1.22 (1.17, 1.27)	< 0.001
** Model 5**	1.00	1.30 (1.20, 1.40)	1.55 (1.41, 1.70)	1.38 (1.13, 1.68)	1.20 (1.15, 1.25)	< 0.001
** Model 6**	1.00	1.27 (1.17, 1.38)	1.51 (1.37, 1.67)	1.36 (1.11, 1.67)	1.19 (1.14, 1.24)	< 0.001
**Female**						
** Abdominal obesity (cases/total, n)**	2635/4891	7020/14181	1457/3046	217/385		
** Model 4**	1.00	1.11 (1.03, 1.19)	1.18 (1.07, 1.30)	1.36 (1.09, 1.69)	1.09 (1.05, 1.14)	< 0.001
** Model 7**	1.00	1.15 (1.07, 1.24)	1.29 (1.17, 1.43)	1.37 (1.10, 1.71)	1.13 (1.08, 1.18)	< 0.001
** Model 8**	1.00	1.14 (1.06, 1.23)	1.27 (1.15, 1.40)	1.32 (1.06, 1.65)	1.12 (1.07, 1.17)	< 0.001

Model 1: adjusted for age (continuous), gender

Model 2: age (continuous), gender, marriage status (married/cohabitation, unmarried or separated or divorced or widowed), education level (primary school and below, middle school, high school, college or university and above), annual household income(< 12,000 CNY, 12,000–19,999 CNY, 20,000–59,999 CNY, 60,000–99,999 CNY, ≥ 100,000 CNY), study area (rural, urban)

Model 3: adjusted as for model 2 plus tobacco use (no smokers, ex-smokers, current smokers), alcohol use (continuous), red and processed meats (continuous), poultry and processed (continuous), fresh vegetables (continuous), fresh fruits (continuous), total energy intake (continuous) and physical activity (continuous)

Model 4: adjusted as for model 1 minus gender

Model 5: adjusted as for model 2 minus gender

Model 6: adjusted as for model 3 minus gender

Model 7: adjusted as for model 2 minus gender, plus menopause

Model 8: adjusted as for model 3 minus gender, plus menopause

### Subgroup analyses between spicy food consumption and abdominal obesity

In subgroup analyses, the strength of the association between spicy food consumption and abdominal obesity was largely consistent across subgroups defined by age, marriage status, education level, annual household income, study area, alcohol status, physical activity, poultry and processed, fresh vegetables, fresh fruits, total energy intake, and menopause status (*P*_*heterogeneity*_ > 0.05). However, there was a significantly stronger association among current smoker (OR = 1.30, 95% CI, 1.15–1.48) than among nonsmokers (OR = 1.23, 95% CI, 1.16–1.48) (*P*_heterogeneity_ < 0.05); red and processed meats intake ≥ 100 g/day group (OR = 1.24, 95% CI, 1.11–1.39) than red and processed meats intake < 100 g/day group (OR = 1.12, 95% CI, 1.06–1.18) (*P*_heterogeneity_ < 0.05) (Table 4).

**Table 4:** ORs (95% CIs) for abdominal obesity associated with consuming spicy food according to participant characteristics

Variables	Abdominal obesity (n, %)	Spicy food consumption		*P*_heterogeneity_
		**Never**	**Yes**	
**Age group (years)**				0.623
** 30–39**	3126 (38.7)	1.00	1.15 (1.00, 1.33)	
** 40–49**	6472 (50.7)	1.00	1.18 (1.07, 1.30)	
** 50–59**	5330 (59.5)	1.00	1.25 (1.12, 1.39)	
** 60–69**	4758 (62.0)	1.00	1.30 (1.17, 1.44)	
** 70–79**	2110 (62.4)	1.00	1.18 (1.02, 1.38)	
**Marriage status**				0.406
** Married/cohabitation**	19331 (53.2)	1.00	1.23 (1.16, 1.30)	
** Unmarried/Separated/divorced/widowed**	2465 (54.6)	1.00	1.23 (1.06, 1.42)	
**Education level**				0.518
** No formal education**	19331 (53.2)	1.00	1.19 (1.04, 1.37)	
** Primary and above**	2465 (54.6)	1.00	1.23 (1.17, 1.30)	
**Annual household income (CNY)**				0.227
** < 60000**	2750 (63.8)	1.00	1.18 (1.11, 1.26)	
** ≥ 60000**	19046 (52.1)	1.00	1.33 (1.21, 1.45)	
**Study area**				0.276
** Rural**	8726 (53.4)	1.00	1.14 (1.06, 1.23)	
** Urban**	13070 (53.3)	1.00	1.30 (1.21, 1.39)	
**Tobacco use**				< 0.001
** Nonsmoker**	15006 (51.7)	1.00	1.23 (1.16, 1.30)	
** Ex-smoker**	1634 (62.3)	1.00	1.25 (1.02, 1.54)	
** Current smoker**	5156 (55.9)	1.00	1.30 (1.15, 1.48)	
**Alcohol use**				0.368
** No drinker**	10110 (52.8)	1.00	1.24 (1.16, 1.33)	
** Occasionally drinker**	6486 (50.5)	1.00	1.21 (1.09, 1.33)	
** Regularly drinker**	5200 (58.6)	1.00	1.17 (1.03, 1.34)	
**Physical activity (MET hours/day)**				0.36
** < 28.14**	11521 (56.4)	1.00	1.25 (1.17, 1.34)	
** ≥ 28.14**	10275 (50.3)	1.00	1.22 (1.13, 1.31)	
**Red and processed meats (g/day)**				0.018
** < 100**	16768 (52.8)	1.00	1.12 (1.06, 1.18)	
** ≥ 100**	5028 (55.3)	1.00	1.24 (1.11,1.39)	
**Poultry and processed (g/week)**				0.359
** < 70**	10817 (53.5)	1.00	1.15 (1.07, 1.23)	
** ≥ 70**	10979 (53.2)	1.00	1.14 (1.06, 1.23)	
**Fresh vegetables and fruits (g/day)**				0.914
** < 400**	21597 (53.3)	1.00	1.14 (1.09, 1.20)	
** ≥ 400**	199 (55.3)	1.00	1.32 (0.79, 2.19)	
**Total energy intake (kcal/day)**				0.184
** < 1644.83**	10483 (51.3)	1.00	1.22 (1.14, 1.30)	
** ≥ 1644.83**	11313 (55.4)	1.00	1.26 (1.17, 1.36)	
**Menopause **^**a**^				0.228
** No**	4605 (38.4)	1.00	1.05 (0.95, 1.17)	
** Yes**	6724 (63.9)	1.00	1.24 (1.13, 1.36)	

Odds ratios were adjusted for age (continuous), marriage status (married/cohabitation, unmarried or separated or divorced or widowed), education level (no formal education, primary and above), annual household income (< 6000 CNY, ≥ 6000 CNY), study area (rural, urban), tobacco use (no smokers, ex-smokers, current smokers), alcohol use (no drinker, occasionally drinker, regularly drinker), physical activity (< 28.14 MET hours/day, ≥ 28.14 MET hours/day), red and processed meats (< 100 g/day, ≥ 100 g/day), poultry and processed (< 70 g/week, ≥ 70 g/week), fresh vegetables fruits (< 400 g/day, ≥ 400 g/day) and total energy intake (< 1644.83 kcal/day, ≥ 1644.83 kcal/day)

^a^ Only in women

## Discussion

To our knowledge, this is the first large study to combine the intake frequency of spicy food and the level of spicy flavor in exploring the association between spicy food consumption and abdominal obesity. In this cross-sectional study, the increase in spicy food intake frequency and strength were associated with the high ratio of abdominal obesity and high WC values. In other words, the consumption of spicy food has positive association with abdominal obesity. Compared with participants who never had spicy food, the participants in the spicy food intake frequency subgroups (1–2 days/week, 3–5 days/week and 6–7 days/week) were positively associated with an increasing risk of abdominal obesity both in males and females. Moreover, compared with participants who never had spicy food, the participants in the spicy food intake strength subgroups (mild, moderate and strong) were positively associated with abdominal obesity in males and females.

Previous intervention studies [20, 31, 40–42] showed a beneficial effect of spicy food consumption on weight management in studies with a small sample size from Western countries. Additionally, a large prospective cohort study [43] of the CHNS data showed that high intake of chilli was inversely associated with the risk of overweight/obesity independent of overall dietary pattern, energy intake and lifestyle factors. In contrast, in the China Kadoorie Biobank (CKB) study [30], the strength and frequency of spicy food consumption was positively associated with adiposity among half a million Chinese adults. Yang et al. found that spicy food consumption was positively associated with adiposity, including both general [44] and abdominal [29] obesity in rural Chinese population, similar to the current study. This positive association may reflect increased palatability of meals including spicy food consumption [20]. The potential reason was spicy food consumption might affect abdominal obesity by increasing energy intake. Because in Chinese cuisines, spicy food are more meat-based rather than vegetable-based [45], with heavy salt and/or oil used for flavor or preservation, such as in hot pot, pickles, typically in Sichuan Basin regions. Additionally, the intake of pungent foods may be accompanied by an increased intake of sweet foods [46], carbohydrate-rich foods [47] to relieve the burning sensation. In this regard, excessive sweet and fat foods intake with spicy foods may also contribute to the positive association of spicy foods intake with obesity. Furthermore, 79.5% of participants consumed spicy food, and the prevalence of weekly spicy food consumption was 51.5% in the present study, much higher than in the CKB population as a whole, in which 42.5% of participants consumed spicy food weekly [30]. But 99.7% of participants in Hunan consumed spicy food weekly, while only 8.8% of participants in Haikou consumed spicy food weekly in the CKB study [30]. This discrepancy reflects geographic distribution of Chinese residents’ preferences for spicy food. Sichuan Basin is located in the southwest of China, and compared with residents living in most other provinces, residents in Sichuan Basin have a preference for a heavier diet rather than bland tastes.

The prospective study using CHNS data showed that high amounts spicy food intake was positively associated with energy intake [43]. Compared with non-spicy food intake group, mean energy intake was greater by more than 200 kcal/day in individuals who consumed more than 50 g/day of chilli. Previous studies [48, 49] have shown that a former exposure to spicy food may attenuate the weight-loss effects. Not only that, numerous studies [50–54] have shown that capsaicin has a potential thermogenic effect, increased energy expenditure associated with capsaicin intake. Therefore, the slimming effects of spicy food consumption on cannot be found in the current study, since participants ate spicy food at different frequencies and levels as a lifetime eating habit. However, capsaicin as the major pungent element in red pepper, and studies of its pharmacological properties on weight management have been inconsistent. An increase in diet-induced thermogenesis and lipid oxidation when high-fat diet is mixed with capsaicin, an increase in energy expenditure after capsaicin intake was shown in study conducted by Andrea et al. [55]. In stark contrast, the human study [56] conducted by Galgani et al. indicated no effect of capsaicin on energy expenditure or lipid oxidation. Thus, the exact underlying mechanism between spicy food and obesity could not be identified and only be speculated.

WC is regarded as the most practical and simplest indicator for evaluating abdominal fat accumulation [57]. In this study, we found that the median WC was in the desirable range for females, but elevated for males. Gender difference in food choice might represent one of the possible reasons for this phenomenon. As females are reported to eat more fruit and vegetables but less high-fat foods than males [58, 59], vegetable-based spicy foods could be good for health; for example, chili pepper is rich in vitamin A, vitamin C and carotenoids. On the other hand, sensitivity to weight-loss effects of spicy food might be modulated by genetic variations [60]. The available studies exploring the associations between spicy food intake frequency and abdominal obesity are still limited. Snitker and collaborators [61] conducted that abdominal obesity decreased to a greater extent in the capsinoid group than in the placebo group, although the mean change in waist girth was not significant. However, the CKB study [30] showed that WC increased with the higher frequency of spicy food intake, which is consistent with the current results. Thus, further studies are required to fully elucidate the effects of spicy food components on abdominal adiposity.

Hot and spicy habits were more common in younger people, but abdominal obesity was higher in older people in our study. The possible is that age is an independent risk factor for abdominal obesity. Over the past several decades, a substantial amount of researches [62–65] have been indicated that the prevalence of abdominal obesity increased with age in both genders. Although spicy habits are more common in younger people, the young people have a faster metabolism and greater energy expenditure than the older people. Additionally, abdominal obesity is defined as abnormal or excessive fat accumulation, which is a long-term effect, and the statistical models have adjusted for age in our analysis.

In our subgroup analyses, significant differences in the association of spicy food consumption with abdominal obesity was observed across strata of tobacco use and red and processed meats consumption, with a stronger positive association among current smokers than nonsmokers, red and processed meats intake ≥ 100 g/day group than red and processed meats intake < 100 g/day group, respectively. Intriguingly, prospective analyses based on 28,773 Henan Rural Cohort Study [29] participants not only showed that the positive association of spicy food consumption was stronger in participants who did smoke than among those who did not smoke. Also, the study indicated that spicy food intake increased the risk of abdominal obesity, and fat energy intake may be a mediator of this association in rural Chinese populations, which is consistent with the current results. Red and processed meats may be an intermediate factor in the association between spicy food and abdominal obesity in our study, and the results need to be approved by large cohort studies or experimental studies.

Several limitations are present in this study. First, as a cross-sectional research, we were unable to reveal a causal association between spicy food consumption and abdominal obesity, and studies with larger sample size and long-term follow-up are needed to validate the associations. Second, for the sake of feasibility, the FFQ used in our study only included 13 crude food groups, not specific food items, the tool might be flawed. However, the food group-based and simplified food questionnaire may be the only feasible way to collect dietary information in a large population-based study. By analyzing the subsample data of 24HDR, we found that the differences between the calculation of total energy intake based on crude food groups and that based on specific food items were small and roughly randomly distributed around zero. Third, the CMEC study is not a nutritional survey, and detailed information on dietary patterns is not available. Therefore, dietary factors, which may be important, were not included in the current analyses. Forth, the energy intake adjusted in our study only included the total energy intake, not specific energy intake types, this might be flawed. Fifth, assessment of spicy food consumption in the current study was self-reported, particularly the strength of spicy food intake was classified according to participants’ subjective evaluations and usual preferences, reporting and recall bias, and measurement error could not be easily ruled out. Moreover, and the variety of chili types might affect the associations, but potential covariates were adjusted and subgroup analyses were conducted to improve the accuracy. Sixth, although multiple established and potential risk factors for abdominal obesity were adjusted for in different models, unknown confounders by other unmeasured or unknown biological and social factors is still possible, which prevented us from judging the association. Finally, the participants in the current study were from two provinces in the southwest region of China, which area eating spicy foods relatively often, and thus the conclusions may not be generalized to other areas in the country. Nevertheless, the results of this study provide some insights into the association between spicy food consumption and abdominal obesity.

## Conclusions

The findings of this large-scare study indicated that spicy food consumption may be a risk factor for abdominal obesity in Chinese adult population. To some extent, we recommend that the residents in the Sichuan Basin may consume spicy food in moderation to reduce the risk of abdominal obesity. However, further multicentre, prospective and long-term follow-up cohort studies are needed to elucidate the mechanisms underlying this association.

## Data Availability

Our study relied on data from the China Multi-Ethnic Cohort Study. The summary dataset used and or analyzed during the current study are available from the corre-sponding author upon reasonable request.

## Abbreviations

CMEC: China Multi-Ethnic Cohort
CKB: China Kadoorie Biobank
CHNS: China health and nutrition survey
WC: Waist circumference
BMI: Body mass index
FFQ: Food Frequency Questionnaire
MET: Metabolic equivalent tasks
ORs: Odds ratios
CI: Confidence interval
TRPV1: Transient receptor potential vanilloid type-1
